# Fracture isolée du petit trochanter chez l'adulte, et si c’était une métastase: à propos d'un cas et revue de la littérature

**DOI:** 10.11604/pamj.2013.16.97.2983

**Published:** 2013-11-15

**Authors:** Hatim Abid, Mohamed El Idrissi, Mohamed Shimi, Abdelhalim El Ibrahimi, Abdelmajid El Mrini

**Affiliations:** 1 Service de chirurgie ostéo-articulaire B4, CHU HASSAN II, Fès, Maroc

**Keywords:** Petit trochanter, fracture, métastase, lesser trochanter, fracture, metastasis

## Abstract

Nous rapportons dans ce travail le cas d'une fracture du petit trochanter sans notion de traumatisme survenue chez une patiente de 85 ans. Il s'agit d'une entité pathologique rare due à une infiltration tumorale de la région trochantérienne souvent de nature métastatique qui peut être méconnue initialement. C’était le cas de notre malade, qui s'est compliquée après 1 mois d’évolution d'une fracture sous trochantérienne. Notre prise en charge thérapeutique était à visée palliative devant des localisations métastatiques multiples.

## Introduction

La fracture arrachement du petit trochanter est une lésion souvent décrite chez l'adolescent sportif dans le cadre d'un exercice violent suite à une contraction brusque du muscle psoas iliaque [1, 2]. Sa survenue chez l'adulte reste exceptionnelle surtout en dehors d'un contexte traumatique, elle renvoie dans ce cas vers un processus tumoral d'origine métastatique le plus souvent [3, 4]. Nous rapportons dans ce travail le cas d'une fracture isolée du petit trochanter sans notion de traumatisme, secondaire à une métastase d'un adénocarcinome thyroïdien.

## Patient et observation

**Figure 1:**
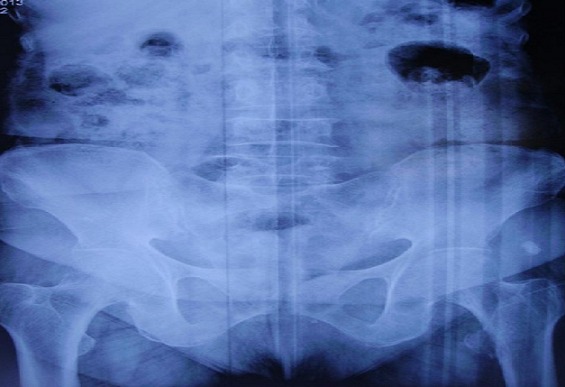
Radiographie de bassin de face objectivant une fracture isolée du petit trochanter gauche

Il s'agit d'une patiente âgée de 85 ans, suivi pour une cardiopathie ischémique sous traitement avec une bonne observance, opérée il y a 10 ans pour un problème thyroïdien non précisé par manque de documents, qui a consulté initialement dans un autre hôpital pour une douleur permanente au niveau de la racine de la cuisse gauche, d'aggravation progressive, sans notion de traumatisme. Le bilan radiologique réalisé comportait des incidences de bassin de face et de la hanche gauche de face en rotation externe mettant en évidence une fracture isolée du petit trochanter non déplacée (Figure 1). Dès lors la patiente a été mise en décharge sous couverture d'un traitement antalgique et anti coagulant.

**Figure 2:**
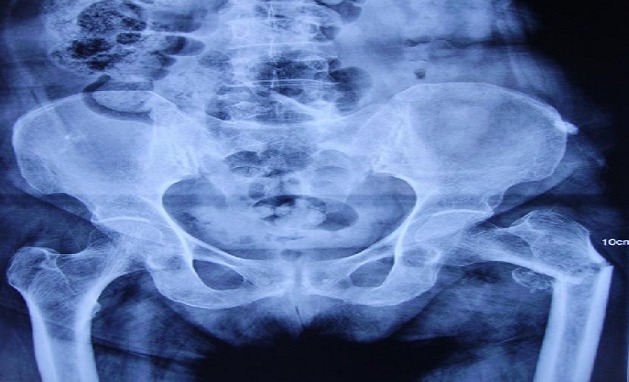
Radiographie de bassin de face objectivant une fracture sous trochantérienne avec ostéolyse manifeste du petit trochanter gauche (après 1 mois)

**Figure 3:**
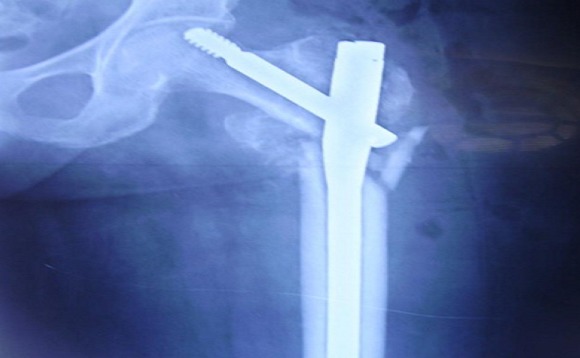
Radiographie de bassin de face en post opératoire immédiat d'une ostéosynthèse par clou gamma long

Un mois après, la patiente s'est présentée à notre consultation pour une aggravation de la douleur à laquelle s'est surajoutée une impotence fonctionnelle totale du membre inférieur gauche survenue suite à une simple tentative d'appui. L'examen clinique avait objectivé une cuisse gauche tuméfiée, déformée en crosse, avec une douleur exquise à la palpation du pli de laine. La mobilisation active et passive était impossible. La radiographie de la hanche gauche de face a mis en évidence en plus de la fracture du petit trochanter, une fracture sous trochantérienne sur une ostéolyse diffuse de l'extrémité proximale du fémur avec une réaction des parties molles en regard (Figure 2). Nous avons procédé à une biopsie osseuse du processus ostéolytique avec la réalisation d'un bilan d'extension. L’étude anatomopathologique du prélèvement est revenue en faveur d'une métastase d'un adénocarcinome thyroïdien. Le scanner thoraco abdomino pelvien a révélé d'autres lésions ostéolytiques de localisation rachidienne. Ainsi nous avons opté pour un traitement palliatif par une ostéosynthèse à l'aide d'un clou gamma long (Figure 3) puis la malade fut transférée en oncologie médicale pour complément de prise en charge.

## Discussion

La fracture isolée du petit trochanter chez l'adulte est une entité lésionnelle rare [3, 4], dans la littérature on dénombre une trentaine de cas. James et al [3] rapportent dans leur travail 15 cas d'atteinte solitaire du petit trochanter sur une série de 295 processus malins de l'extrémité supérieure du fémur, l'origine métastatique constituait 60% des cas pour 70% retrouvé dans la littérature. Bertin et Phillips [5, 6] rapportent chacun 4 cas de métastases du petit trochanter responsable de son arrachement, la tumeur primitive était un adénocarcinome prostatique dans 2 cas, thyroïdien, pancréatique et colique dans 1 cas puis d'origine indéterminée dans un seul cas. En ce qui concerne les tumeurs primitives du petit trochanter, les cas rapportés séparément par Afla, James et Jake ne représentent que 30% de l'ensemble des tumeurs atteignant cette localisation avec une prédominance du myélome et des chondrosarcomes [3, 4, 7].

Sur le plan radiologique, la radiographie standard de la hanche de face permet de poser le diagnostic d'une fracture du petit trochanter. Dans ce sens James et Dimon [3, 8] signalent l'importance de l'incidence de profil pour ne pas méconnaitre une fracture non déplacée, elle permet aussi de visualiser l'atteinte lytique de la base du petit trochanter. Concernant l'IRM elle est désormais indispensable pour la plupart des auteurs [3, 4, 6, 7] dans le but d'apprécier l'infiltration tumorale des structures de voisinage et de préciser les limites de résection. En matière de bilan d'extension à distance, la majorité des auteurs [4, 6, 7] tendent vers la scintigraphie au technétium 99 au dépend du scanner dont on s'en sert de plus en plus pour la réalisation de biopsie radio guidée.

Pour ce qui est de la prise en charge thérapeutique, elle dépend du stade évolutif de la tumeur. L'attitude est palliative dans les stades avancés [5, 9], alors que la résection carcinologique suivie d'un remplacement prothétique est préconisés devant une métastase unique ou un primitif bien limité [5]. De ce fait nous avons opté pour un traitement palliatif chez notre malade.

## Conclusion

La fracture isolée du petit trochanter chez l'adulte en dehors d'un contexte traumatique ou après un traumatisme mineur constitue une entité pathologique rare. Elle doit faire penser à une infiltration tumorale souvent d'origine métastatique dont il faut rechercher la tumeur primitive. Le traitement est fortement conditionné par le stade évolutif de la maladie.
